# Genome-wide identification of altered RNA m^6^A profiles in vascular tissue of septic rats

**DOI:** 10.18632/aging.203506

**Published:** 2021-09-10

**Authors:** Zhu-Jun Shen, Ye-Chen Han, Mu-Wen Nie, Yi-Ning Wang, Ruo-Lan Xiang, Hong-Zhi Xie

**Affiliations:** 1 Department of Cardiology, Peking Union Medical College Hospital, Chinese Academy of Medical Sciences and Peking Union Medical College, Beijing 100730, China; 2 Department of Physiology and Pathophysiology, Peking University School of Basic Medical Sciences, Beijing 100191, China

**Keywords:** N6-methyladenosine, sepsis, aorta

## Abstract

Sepsis is the leading cause of death in hospital intensive care units. In light of recent studies showing that variations in N^6^-methyladenosine (m^6^A) levels in different RNA transcripts influence inflammatory responses, we evaluated the m^6^A profiles of rat aortic mRNAs and lncRNAs after lipopolysaccharide (LPS)-induced sepsis. LC-MS-based mRNA modification analysis showed that global m6A levels were significantly decreased in aortic tissue of rats injected intraperitoneally with LPS. This finding was consistent with downregulated expression of METTL3 and WTAP, two members of the m^6^A writer complex, in LPS-exposed aortas. Microarray analysis of m^6^A methylation indicated that 40 transcripts (31 mRNAs and 9 lncRNAs) were hypermethylated, while 223 transcripts (156 mRNAs and 67 lncRNAs) were hypomethylated, in aortic tissue from LPS-treated rats. On GO and KEGG analyses, ‘complement and coagulation cascades’, ‘transient receptor potential channels’, and ‘organic anion transmembrane transporter activity’ were the major biological processes modulated by the differentially m^6^A methylated mRNAs. In turn, competing endogenous RNA network analysis suggested that decreased m^6^A levels in lncRNA-XR_343955 may affect the inflammatory response through the cell adhesion molecule pathway. Our data suggest that therapeutic modulation of the cellular m^6^A machinery may be useful to preserve vascular integrity and function during sepsis.

## INTRODUCTION

Organ damage and septic shock are two major contributing factors to the high mortality associated with sepsis, a syndrome characterized by a disproportionate host immune response to infectious injury [1–3]. The sepsis-related mortality rate is exacerbated by septic shock, which causes circulatory failure, leading to organ hypoperfusion and ultimately organ failure. Adequate organ perfusion is largely dependent on normal diastolic blood pressure, which is influenced by cardiac output and thickness and elasticity of the aortic wall and peripheral vessels. Although aortic dysfunction is known to aggravate sepsis progression, the mechanisms responsible for aortic injury during sepsis remain insufficiently characterized [4].

Recent studies have uncovered approximately 100 different chemical modifications of RNAs that potentially affect, without altering their specific sequences, their folding and structure, stability, and function. The N^6^-methyladenosine (m^6^A) modification is the most abundant internal modification in mRNAs and occurs also in most non-coding RNA species, including long non-coding RNAs (lncRNAs). Accumulating evidence indicates a relevant role for m^6^A methylation in several gene expression steps, affecting transcript stability, export, splicing, and translation [5, 6]. The m^6^A modification is reversible and depends on the activity of methylases, demethylases, and adapter proteins termed m^6^A “writers”, “readers”, and “erasers” that mediate respectively the methylation, functional properties, and demethylation of the target RNAs [7, 8].

Changes in the m^6^A profile of various RNAs were shown to modulate many physiological and biological processes and to contribute to the pathogenesis of cardiovascular diseases [9, 10]. However, there is scarce information about m^6^A alterations in mRNAs and lncRNAs during sepsis, particularly in vascular tissues [11]. Therefore, we performed genome-wide screening of m^6^A modifications in lncRNAs and mRNAs from aortic tissues of septic rats and inferred, through bioinformatics analyses, the potential implications of the observed changes. Our findings may help identify new therapeutic targets to reduce the morbidity and mortality associated with septic syndromes.

## RESULTS

### Sepsis decreases global mRNA m^6^A levels in the rat aorta

**Figure 1:**
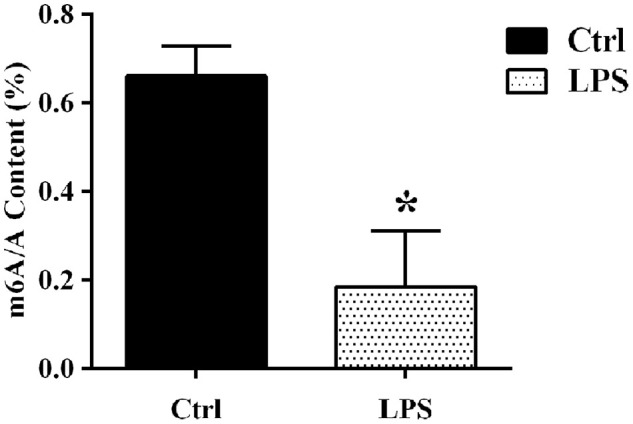
**LC-MS-based analysis of sepsis-induced alterations in m^6^A levels in aortic mRNAs.** Ctrl, control.

To evaluate potential changes in the m^6^A profile of aortic RNA species following sepsis, an intraperitoneal injection of lipopolysaccharide (LPS) was applied to rats to establish a sepsis model. Twenty-four h after LPS injection, mean arterial pressure (MAP) decreased by 30% relative to baseline. There was no significant change in MAP in rats injected with saline (control). The aortic tissues were carefully removed from rats after 24 hours of LPS/saline injection. LC-MS-based mRNA modification analysis was next used to detect global m^6^A levels in aortic mRNA. The results showed that the abundance of m^6^A sites was significantly decreased in the LPS group compared with the control group (Figure 1).

### Microarray-based analysis of differentially m^6^A-modified mRNAs and lncRNAs

**Figure 2:**
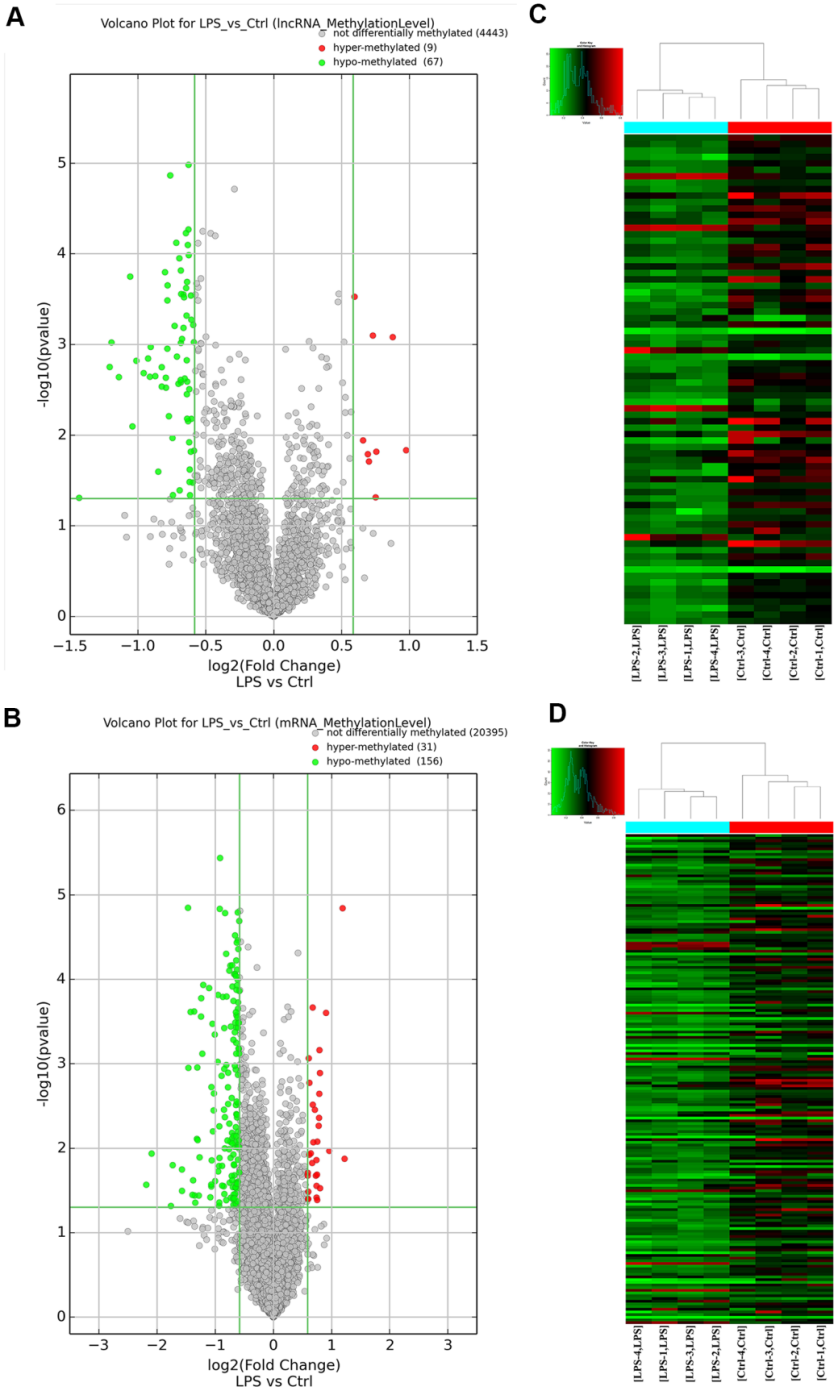
**Overview of the m^6^A methylation map in aortic tissues.** (**A**) Scatter plots showing differentially methylated lncRNAs. (**B**) Scatter plots showing differentially methylated mRNAs. (**C**) Hierarchical clustering analysis of lncRNAs with significantly altered m^6^A levels. (**D**) Hierarchical clustering analysis of mRNAs with significantly altered m^6^A levels. Ctrl, control.

After antibody-based m^6^A labeling and immunoprecipitation of total aortic RNA, microarray analysis revealed that a total of 263 transcripts (187 mRNAs and 76 lncRNAs) in the LPS group had significantly altered (fold change > 1.5; *P* < 0.05) m^6^A abundance compared with the control group. Among these differentially m^6^A-modified transcripts, 84.8% (156 mRNAs and 67 lncRNAs) showed downregulated m^6^A levels, whereas the remaining 15.2% (31 mRNAs and 9 lncRNAs) showed instead upregulated m^6^A levels. Based on m^6^A fold-change ranking, information on 19 lncRNAs (the 9 hypermethylated and the top 10 hypomethylated ones) and 20 mRNAs (top 10 hypo- and hypermethylated mRNA transcripts) is provided in Supplementary Tables 1, 2. The mRNAs and lncRNAs with significantly altered m^6^A profiles were then lined up for cluster analysis according to the similarity of their m^6^A methylation levels and the closeness of their relationship. Variations in m^6^A patterns between the two groups are depicted in Figure 2 using volcano plots.

### GO and KEGG analysis of differentially methylated mRNAs

**Figure 3:**
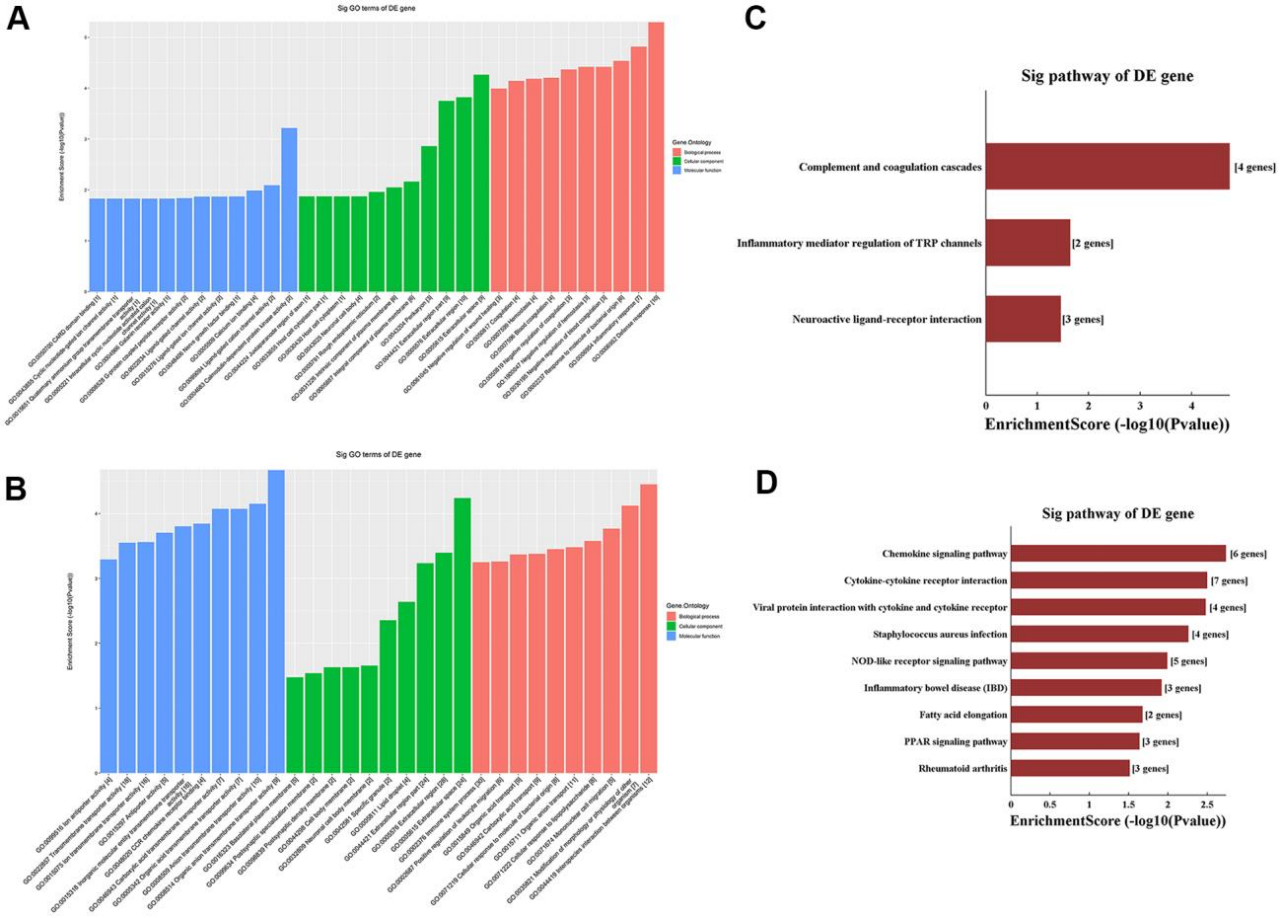
**Functional enrichment analysis of differentially methylated mRNAs.** (**A**) Top ten GO terms for hypermethylated mRNAs. (**B**) Top ten GO terms for hypomethylated mRNAs. (**C**) Top ten KEGG pathways for hypermethylated mRNAs. (**D**) Top ten KEGG pathways for hypomethylated mRNAs.

We performed Gene Ontology (GO) and Kyoto Encyclopedia of Genes and Genomes (KEGG) analyses of the 31 m^6^A hypermethylated and the 156 m^6^A hypomethylated mRNAs obtained by microarray. Among the enriched GO terms for the 31 m^6^A hypermethylated mRNAs, ‘defense response’ in BP, ‘extracellular space’ in CC, and ‘calmodulin-dependent protein kinase activity’ in MF had the highest enrichment scores (Figure 3A, Supplementary Table 3). In turn, the most enriched GO terms for the 156 m^6^A hypomethylated mRNAs included ‘interspecies interaction between organisms’ in BP, ‘extracellular space’ in CC, and ‘organic anion transmembrane transporter activity’ in MF (Figure 3B, Supplementary Table 4). Meanwhile, KEGG analysis of the 31 m^6^A hypermethylated mRNAs revealed significant enrichment in ‘complement and coagulation cascades’, ‘inflammatory mediator regulation of transient receptor potential (TRP) channels’, and ‘neuroactive ligand-receptor interaction’ pathways (Figure 3C). Among the 156 m^6^A hypomethylated mRNAs, nine pathways were enriched, with top scores retrieved for ‘chemokine signaling pathway’ and ‘cytokine-cytokine receptor interaction’ (Figure 3D, Supplementary Table 5).

### M^6^A single-base site qPCR with MazF treatment

**Figure 4:**
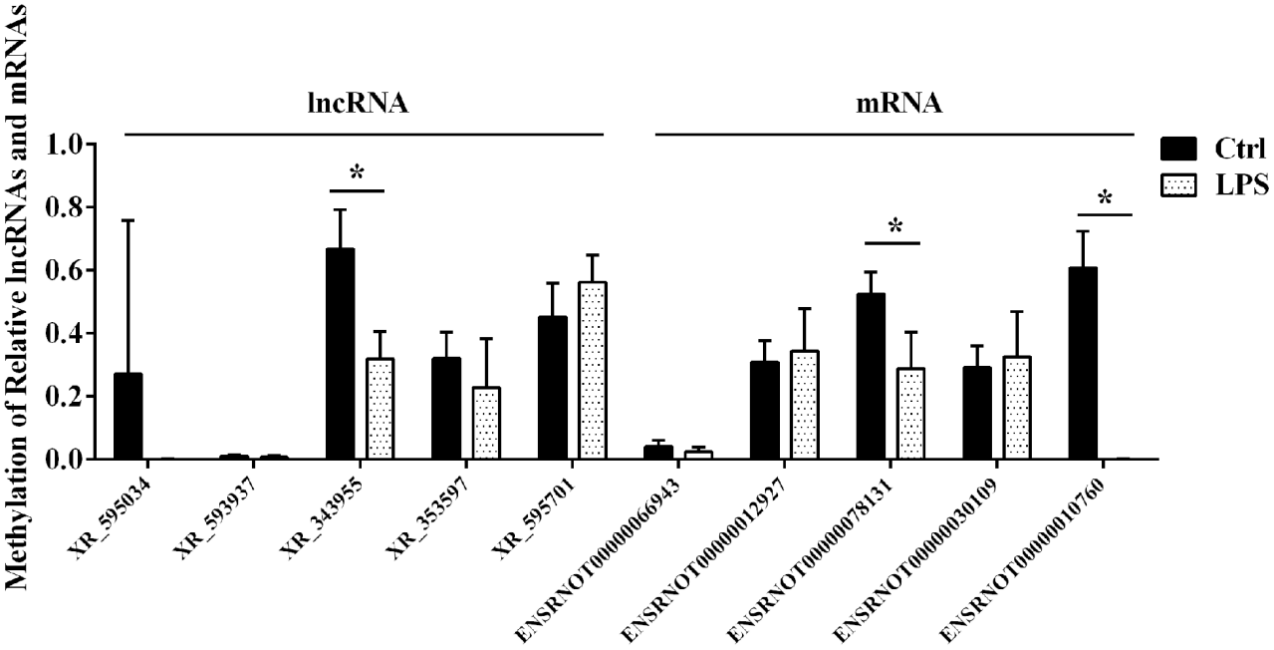
**Confirmatory analysis of microarray results.** M^6^A single-base site qPCR was used to validate the top five differentially methylated aortic lncRNAs and mRNAs identified through microarray in the LPS and Ctrl groups. Ctrl, control.

Based on information in the SRAMP database, we performed motif analysis of m^6^A ACA sequences in the differentially m^6^A methylated lncRNAs and mRNAs detected by microarray and verified the presence of m^6^A-modified sites by qRT-PCR using the MazF enzyme treatment method. Five lncRNAs and five mRNAs with high-confidence m^6^A-modified sites were thus selected for validation using m^6^A single-base site qPCR (Table 1). Consistent with microarray data, this analysis confirmed significant downregulation of m^6^A in the lncRNA XR_343955. In contrast, no significant alterations in m^6^A levels were detected for the other four candidate lncRNAs. Among the five mRNAs considered, significant m^6^A downregulation was detected for both ENSRNOT00000010760 (consistent with microarray data) and ENSRNOT00000078131 (contrary to microarray results), while no significant alterations were observed in the other three mRNAs (Figure 4).

**Table 1:** Targeted lncRNAs and mRNAs from microarray predicted by SRAMP.

**GeneSymbol**	**Transcript ID**	**Transcript type**	**RNA length**	**Position**	**Regulation**	**Fold change**	**P-value**
LOC103693543	XR_595034	lncRNA	1931	1359	hyper	1.835972714	0.000829838
LOC103690224	XR_593937	lncRNA	1386	914	hypo	0.436711298	0.000951609
LOC102554997	XR_343955	lncRNA	1787	1482	hypo	0.60799908	7.56021E-05
Leprel2	XR_353597	lncRNA	2222	1781	hypo	0.630825555	0.000655905
LOC103693720	XR_595701	lncRNA	1332	1021	hypo	0.639273802	0.000238771
Tnfrsf26	ENSRNOT00000066943	protein_coding	2294	416	hyper	2.335986528	0.013354003
Fibcd1	ENSRNOT00000012927	protein_coding	4057	2519	hyper	1.587756196	0.014968993
Kng1	ENSRNOT00000078131	protein_coding	1905	1377	hyper	1.532870761	0.011963925
Colgalt2	ENSRNOT00000030109	protein_coding	1875	1730	hypo	0.411387391	0.03636713
Mettl7b	ENSRNOT00000010760	protein_coding	1264	654	hypo	0.415309496	0.012844316

### ceRNA network construction and functional enrichment analysis of target mRNAs

**Figure 5:**
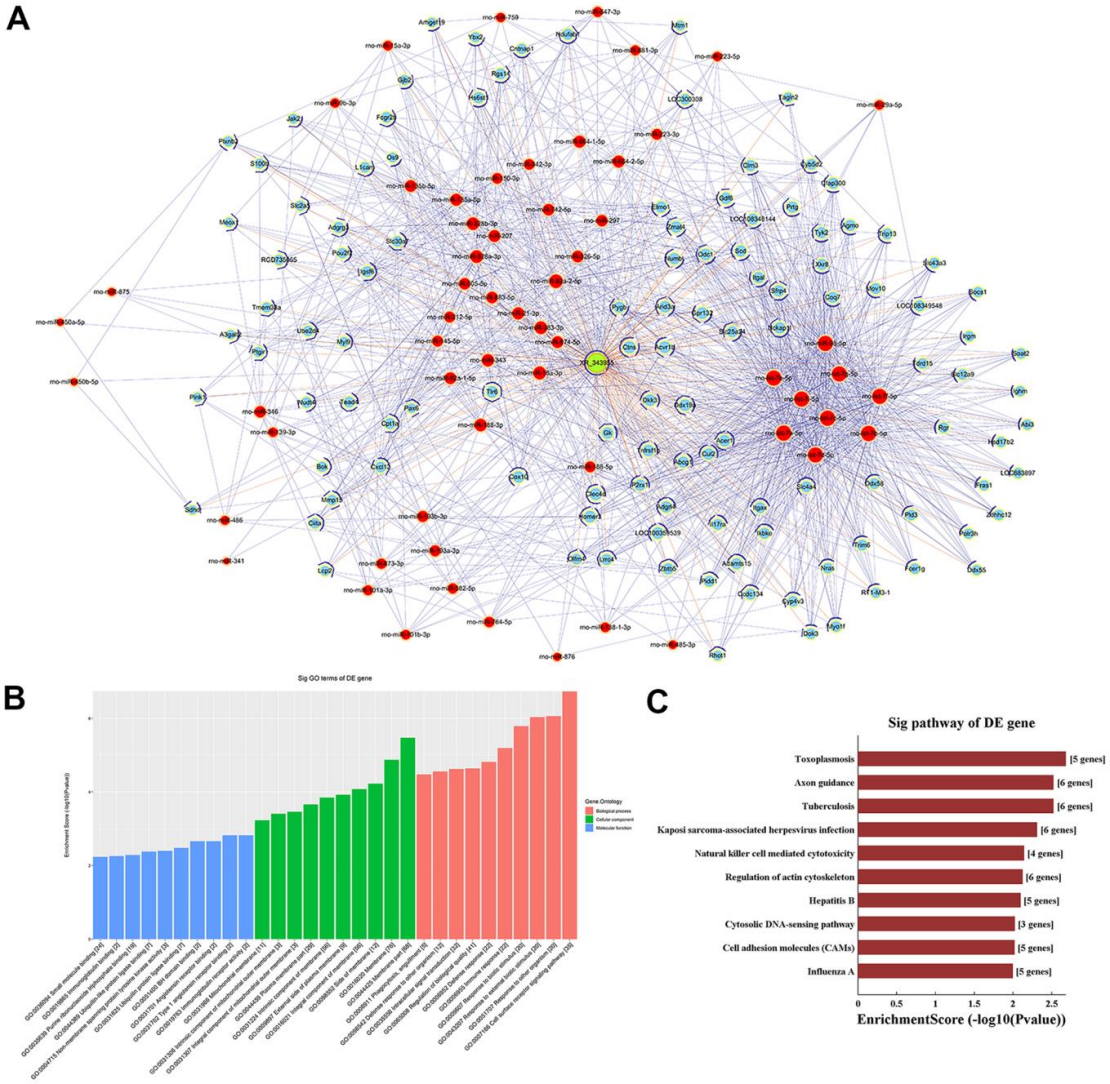
**LncRNA-XR_343955-based ceRNA network.** (**A**) XR_343955-associated ceRNA network. Red circles represent miRNAs, blue circles represent mRNAs, and green circles represent lncRNAs. (**B**) Histogram representation of GO functional classification of predicted mRNAs. (**C**) Histogram representation of KEGG pathway enrichment for predicted mRNAs.

Based on m^6^A single-base site qPCR results, we constructed a competing endogenous RNA (ceRNA) network to identify putative miRNAs and mRNAs regulated by lncRNA-XR_343955. By confining the number of miRNA-IDs to 1000, 59 miRNA binding sites and 118 targeted mRNAs were predicted (Figure 5A). To assess the potential biological functions of lncRNA XR_343955, the 118 mRNAs thus retrieved were analyzed with GO and KEGG. For this mRNA set, the GO terms with the highest enrichment were ‘cell surface receptor signaling pathway’ in BP, ‘membrane part’ in CC, and ‘immunoglobulin receptor activity’ in MF (Figure 5B). In turn, KEGG analysis of the 118 mRNAs indicated cumulative enrichment in 27 pathways, of which the top 10 are shown in Figure 5C.

### Expression analysis of m^6^A effector proteins

**Figure 6:**
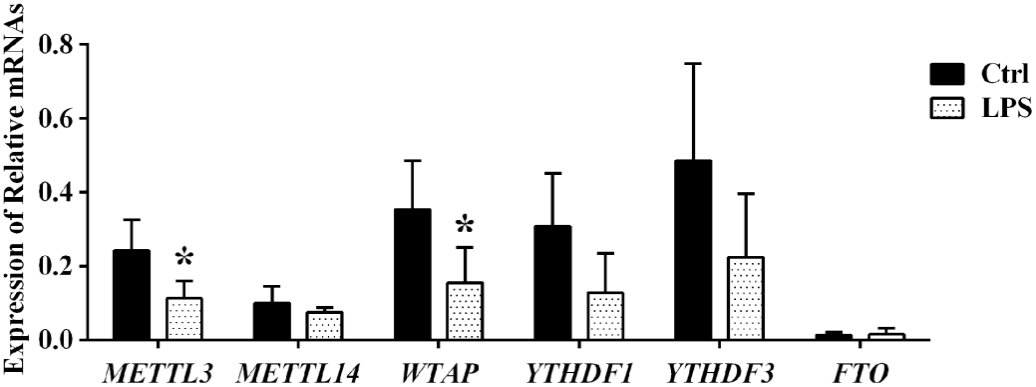
Expression analysis of m^6^A effector proteins by qPCR.

We next used qRT-PCR to detect mRNA levels of key proteins regulating the m^6^A modification, namely m^6^A writers (METTL3, METTL14, and WTAP), readers (YTHDF 1 and YTHDF 3), and an eraser (FTO), in aortic samples from control and LPS-treated rats. Compared to control, in the LPS group the expression of both METTL3 and WTAP was significantly downregulated, while that of YTHDF 1, YTHDF 3, METTL14, and FTO remained essentially unchanged (Figure 6). These data suggest that the predominant m^6^A demethylation pattern observed for aortic RNA during sepsis is due, at least in part, to downregulation of the METTL3 methylase and the adapter protein WTAP.

## DISCUSSION

The m^6^A modification of eukaryotic RNA has a wide-ranging effect on RNA homeostasis [9]. Therefore, alterations in RNA m^6^A methylation status can lead to cell dysfunction and disease [12]. Recent studies have revealed that the m^6^A modification not only has a strong and intricate relationship with cardiovascular disease but may regulate also the inflammatory response arising during different physiopathological conditions [13, 14]. Although the clinical significance of m^6^A profiling in sepsis patients has been recently suggested [11], there is a clear need for studies addressing the specific m^6^A alterations occurring in tissues and organs affected by sepsis. More extensive data exist for the role of the m^6^A modification in cardiovascular disease. Using clinical human samples, primary cardiomyocyte cultures, and preclinical pig and mouse models, Prabhu et al. found that increased levels of m^6^A during ischemia/hypoxia, resulting from downregulated FTO expression, are associated with impaired cardiomyocyte contractile function [13]. Consistently, Song et al. showed increased representation of m^6^A sites in mouse heart mRNAs following ischemia/reperfusion. Interestingly, this phenomenon was associated with increased expression of METTL3, a component of the m^6^A writer complex, and autophagy activation [15]. The impact of abnormal m^6^A expression patterns on inflammation was highlighted by Zhang et al., who reported that the m^6^A reader protein YT521-B homology domain family 2 (YTHDF2) activates the LPS-induced inflammatory response in preosteoblast MC3T3-E1 cells by regulating the MAPK signaling pathway [16]. In turn, Feng et al. reported that knockdown of METTL3 inhibited inflammation by allowing the expression of an alternatively spliced isoform of MyD88 in human dental pulp cells [17].

To explore potential changes in the m^6^A methylation profiles of aortic RNA species during sepsis-induced vascular injury, we established a sepsis model by intraperitoneally injecting LPS into Wistar rats. We found that both global levels of m^6^A and the expression of m^6^A writer complex proteins were significantly decreased in aortic tissue of LPS-treated rats. These results suggest that sepsis-induced changes in the m^6^A profile of aortic RNA species may be related to the vascular injury associated with septic syndromes.

GO and KEGG analysis of the minor fraction of mRNAs with upregulated m^6^A levels revealed their enrichment in coagulation processes. Although coagulation and inflammation represent basic host responses against infection, lack of resolution of these processes may cause damage to host cells and tissues. In sepsis, increased coagulation activity and decreased fibrinolysis caused by inflammation lead to fibrin deposition in the microcirculation. This in turn causes disseminated intravascular coagulation (DIC), ultimately leading to organ dysfunction [18, 19]. In patients with sepsis complicated by severe coagulopathy and/or DIC, organ dysfunction and mortality are significantly increased [20]. Over the past few decades, diverse anticoagulants such as serine protease inhibitors, recombinant human activated protein C, and tissue factor pathway inhibitor have been used as adjunctive therapies for patients with sepsis. However, two meta-analyses, conducted in 2003 and 2016, showed that anticoagulation was not beneficial in reducing mortality and was in turn associated with increased bleeding complications [21, 22]. Aiding the search for safer and more effective anti-DIC therapies, our results suggest that global or mRNA-specific therapeutic modulation of m^6^A methylation dynamics may be useful to regulate hemostasis and prevent or attenuate sepsis-induced DIC and organ failure.

GO analysis of aortic mRNAs with upregulated m^6^A levels demonstrated that several transcripts, many of which encode cation channels, were enriched in ion channel activity in the BP category. In turn, GO analysis of mRNAs with downregulated m^6^A expression demonstrated that numerous transcripts, many of those encoding proteins with organic anion transmembrane transporter activity, were associated with ion transmembrane transporter activity in the MF category.

The cations related to vascular function are primarily calcium (Ca^2+^) ions. Intracellular Ca^2+^ plays an important role in the modulation of vascular smooth muscle cell (VSMC) elasticity by affecting contraction and cell signaling [23–26]. In vascular endothelial cells (VECs), Ca^2+^ levels also influence the production of nitric oxide, a key regulator of vasoconstriction and diastolic function [27, 28]. The large number of cation channel mRNAs with increased m^6^A abundance detected in aortic tissue during sepsis may be partly responsible for the abnormal intracellular Ca^2+^ dynamics associated with septic vascular injury. Our KEGG analysis of mRNAs with upregulated m^6^A modification revealed in turn significant enrichment in the pathway related to inflammatory mediator regulation of TRP channels. This finding is consistent with the results of the GO analysis, since several members of the TRP protein family are Ca^2+^-selective channels. TRP channels are highly sensitive to various physical and chemical stimuli, and inflammation can lead to an influx of a large amount of Ca^2+^ into cells by activating TRP channels. In primary human osteoarthritis fibroblast-like synoviocytes, LPS stimulation leads to increased expression of TRP ankyrin 1 (TRPA1), enhanced TRPA1-mediated Ca^2+^ influx, and synthesis of pro-inflammatory factors [29]. In acute lung injury, TRP vanilloid 4 (TRPV4)-dependent Ca^2+^ influx contributes to LPS-induced macrophage activation, a process associated with the calcineurin-NFATc3 pathway [30]. These studies suggest that during inflammation the intracellular Ca^2+^ concentration can be affected via TRP channel activity.

Based on our high-throughput sequencing results, we speculate that increased m^6^A levels in mRNAs coding for cation channels, particularly TRP channels, contribute to dysregulated Ca^2+^ dynamics in sepsis and impaired VEC and VSMC function.

Our results showed decreased m^6^A levels in several aortic mRNAs related to organic anion transmembrane transporter activity. The anions involved in the regulation of vascular function are primarily chloride ions. Although the volume of healthy cells remains relatively stable, cell volume changes often occur during physiological and pathophysiological processes such as proliferation, migration, differentiation, and apoptosis [31]. When the cells swell, Cl^-^ together with Na^+^ and water flow out through the "Cl^-^ channels", "Ca^2+^-activated Cl^-^ channels", or "transporters", and a swollen cell can return to normal size. During sepsis, inflammatory factors cause the swelling and dysfunction of VSMCs and VECs, and changes in the Cl^-^ channels affect water drainage and vascular function [32, 33]. Our analysis of microarray data shows m^6^A levels of solute carrier family (SLC) gene were significantly decreased in aortic tissue during sepsis. The phylogenetically ancient SLC26 gene family encodes multifunctional anion exchangers and anion channels transporting a broad range of substrates, including Cl^-^, HCO^3-^, sulfate, oxalate, I^−^, and formate. It has been reported that SLC 26 member 7 (SLC26A7) was identified as a chloride–bicarbonate anion exchanger and/or as a Cl^-^ channel in the kidney and stomach [34, 35], whose gene mutations cause congenital deafness and dyshormonogenic goiter [36]. Alterations in Cl^-^ channels can affect intracellular water content, vascular tone and arterial blood pressure. These findings may be related to the impairment of vascular function in sepsis. The m^6^A modification provides a new research direction for vascular function protection.

Our results showed also significantly altered m^6^A levels in 76 aortic lncRNAs after LPS-induced sepsis. Although most studies on the m^6^A modification have focused on its regulatory role in mRNA function, recent evidence suggests that the m^6^A methylation regulates also the synthesis and function of lncRNAs [37]. Consistent with our microarray results, single-base site qPCR confirmed significant downregulation of m^6^A sites in lncRNA XR_343955 in the aorta of LPS-treated rats. GO and KEGG analyses of 118 predicted mRNAs detected via the ceRNA network for XR_343955 revealed significant enrichment of these transcripts in pathways involving cell adhesion molecules (CAMs). CAMs such as ICAM-1, E-selectin, and VCAM-1 play key roles in the vascular inflammation process by mediating the adhesion of circulating leukocytes to the vascular endothelium before extravasation into the vascular wall [38–41]. During sepsis, secretions of pro-inflammatory HMGB1 by endothelial cells can upregulate the expression of CAMs to promote the inflammatory response by recruiting leukocytes [42, 43]. Therefore, inhibiting the expression of CAMs in VECs is considered a promising treatment for vascular inflammatory diseases. Our results suggest that XR_343955 may regulate CAMs through a ceRNA mechanism, and such capacity may be in turn influenced by sepsis-related changes in XR_343955’s m^6^A profile.

In mammalian cells, dynamic and reversible m^6^A modification is governed by the activity of m^6^A methyltransferases and adapter proteins (i.e. m^6^A writers: METTL3, METTL14, and WTAP) and m^6^A demethylases (i.e. m^6^A erasers: FTO and ALKBH5), which install and remove, respectively, m^6^A marks in target RNAs. Another regulatory layer is further established by specific RNA-binding proteins (i.e. readers: YTHDF1/3) which bind to the m^6^A motif to affect RNA function. Using qRT-PCR, we detected that the expression of METTL3 and WTAP in the aorta was significantly downregulated during sepsis, while that of YTHDF 1, YTHDF 3, METTL14, and FTO did not change significantly. Downregulation of the m^6^A writers METTL3 and WTAP in aortic tissue during sepsis is thus consistent with both LC-MS data, which suggested a decrease in global m^6^A demethylase activity for the mRNA pool, and with microarray results, which showed decreased m^6^A levels in 84.8% of the mRNA/lncRNA transcripts with significantly altered m^6^A status. Dysregulated expression of m^6^A effectors has shown to contribute to cancer pathogenesis. For example, upregulated METTL3 expression was shown to promote gastric cancer and hepatocellular carcinoma progression by promoting, respectively, epithelial to mesenchymal transition and posttranscriptional silencing of SOCS2 [44, 45]. Therefore, it is plausible that downregulation of METTL3 and WTAP may modulate aortic damage during sepsis.

The m^6^A modification is an important modification of RNA, which has received extensive attention. However, it is difficult to quickly and efficiently study the relationship between m^6^A modification of RNA and diseases by relying on traditional biological experiments. Microarray analysis is a powerful tool that can reveal the differential expression profile of m^6^A-related RNAs underlying specific phenotypic differences. In addition, bioinformatics analysis methods and computational models could be effective ways to identify potential RNAs functions and RNA–disease associations. This might greatly decrease the time and cost of biological experiments. In our study, microarray analysis was performed, followed by bioinformatics analysis using a computer model. This can help researchers quickly and efficiently identify m^6^A-related RNAs associated with damage of aorta tissues during sepsis. However, our study has some potential limitations. First, differentially expressed m^6^A-related RNAs are mainly detected in the aorta tissues. Therefore, it is impossible to distinguish whether these changes occurs in vascular smooth muscle cells or vascular endothelial cells. Second, the sample size of microarray analysis was relatively small. Last, there were variations in differentially m^6^A methylated lncRNAs and mRNAs detected by microarray and m^6^A single-base site qPCR, which may be due to the methodological differences.

In conclusion, we detected differential abundance of m^6^A bases in numerous rat aortic mRNAs and lncRNAs, as well as altered expression of m^6^A writer proteins, following LPS-induced sepsis. GO and KEGG analyses indicated that the differentially m^6^A-modified mRNAs were mainly related to ‘complement and coagulation cascades’, ‘TRP channels’, and ‘organic anion transmembrane transporter activity’. In turn, ceRNA network analysis suggested the involvement of lncRNA-XR_343955 in the inflammatory response through regulation of CAM-related pathways. These findings suggest that therapeutic modulation of the cellular m^6^A machinery may be valuable to treat coagulation defects, attenuate inflammatory responses, and preserve vascular integrity in the setting of sepsis.

## MATERIALS AND METHODS

### Animals

Eight-week-old male Wistar rats (250–350 g) were purchased from Charles River Laboratories (Beijing, China). Experimental procedures involving the use of animals complied with both ARRIVE guidelines (Consort Group, 2010) as well as with relevant national laws on animal protection, and the protocol was approved by the Ethics Committee on Animal Research at Peking University Health Science Center (Ethics No. LA2020343). Following adaptation to standard laboratory conditions for one week, the experimental rats were randomly allocated to one of two groups: the LPS-induced sepsis group (n = 4) received an intraperitoneal injection of 10 mg/kg LPS (*Escherichia coli* 055:B5; Sigma-Aldrich, USA; 5 mg of LPS dissolved in 1 mL of 0.9% saline); the control group (n = 4) was intraperitoneally injected with 0.9% saline (2 ml/kg). MAP was noninvasively measured 24 h after the LPS/saline injection. The aortic tissue (n = 4/group) were carefully removed from the anaesthetized rats, immediately frozen in liquid nitrogen and stored at -80° C until analysis.

### RNA extraction and quality control

Total RNA from aortic tissues (n = 4 per group) was isolated and assessed as previously described [46]. Briefly, total RNA was isolated from the aortic tissues using TRIzol Reagent according to the manufacturer’s instruction (Invitrogen, USA). The quantity and purity of the total RNA samples were measured by a NanoDrop ND-1000 (ThermoFisher, USA).

### LC-MS/MS-Based mRNA m^6^A modification detection

The mRNA was isolated and purified from total RNA using the NEBNext Poly(A) mRNA Magnetic Isolation Module (NEB, USA) and a Qubit RNA HS Assay kit (Thermo Fisher, USA). The mRNA was then hydrolyzed into single dephosphorylated nucleosides with an enzyme mix. The pretreated nucleoside solution was deproteinized using a Sartorius 10,000-Da MWCO spin filter. LC-MS/MS analysis was performed on an Agilent 6460 QQQ mass spectrometer with an Agilent 1260 HPLC system in multi-reaction monitoring (MRM) detection mode (n = 4/ group).

### Detection of m^6^A-modified mRNAs and lncRNAs by microarray hybridization

Sample preparation and microarray hybridization procedures were based on Arraystar’s standard kit assays and protocols (Arraystar, USA). In this study, up to 27770 mRNAs and 10582 lncRNAs could be detected in a single array using the probes contained in Arraystar’s Rat mRNA and lncRNA Epitranscriptomic Array (m^6^A). In brief, purified total RNA from aortic tissue of LPS-treated and control rats was immunoprecipitated with polyclonal anti-m^6^A antibody (Cat 202003Synaptic Systems, USA). The m^6^A-tagged RNAs were eluted from the immunoprecipitated (IP) magnetic beads and the unmodified RNAs were eluted from the supernatant (Sup). The IP and Sup RNA fractions were then labeled with Cy5 and Cy3, respectively, as cRNAs in separate reactions using the Arraystar Super RNA Labeling Kit. The labeled cRNAs were then combined and hybridized onto an Arraystar Rat mRNA and lncRNA Epitranscriptomic Microarray (4x44K, Arraystar, USA) and scanned in two-color channels with an Agilent Scanner G2505C (Agilent, USA).

### Microarray data analysis

Agilent Feature Extraction software (version 11.0.1.1) was used to analyze the acquired array images. The raw intensities of the IP (immunoprecipitation, Cy5-labeled) and Sup (supernatant, Cy3-labeled) RNA fractions were normalized using the average of the log2-scaled spike-in RNA intensities. Following spike-in normalization, the probe signals that displayed present (P) or marginal (M) QC flags in at least 4 out of 8 samples were retained as “All Targets Values” in an Excel sheet for further “m^6^A methylation level” analyses. The “m^6^A methylation level” was calculated as follows:

$$\text{\%Modified}=\;\frac{\text{modified RNA}}{\text{Total RNA}}\;=\;\frac{\text{IP}}{\text{IP}+\text{Sup}}\;$$

$$\frac{{\text{IP}}_{Cy5\;normalized\;intensity}}{{\text{IP}}_{Cy5\;normalized\;intensity}+{\text{Sup}}_{Cy3\;normalized\;intensity}}$$

Differentially m^6^A-methylated RNAs between two comparison groups were identified by filtering by fold change (≥ 1.5) and statistical significance (*P* < 0.05) thresholds (n = 4 for each group).

### M^6^A single-base site quantitative real-time PCR

The methylated lncRNAs and mRNAs were quantified by m^6^A single-base site qPCR with the MazF treatment method according to KangChen’s standard protocols (KangChen Biotech., China). In brief, the MazF treatment mixture was dispensed into a 10 μl volume with 1 μg of total RNA from each aortic tissue sample (n = 4 per group) and 20 U mRNA interferase-MazF (Takara, Japan) at 37° C for 30 min. One microgram of nondigested total RNA was reserved. The digested mRNA and the nondigested total RNA samples were subjected to reverse transcription using SuperScript^™^ III Reverse Transcriptase (Invitrogen, USA) for qPCR with a QuantStudio5 Real-time PCR System (Applied Biosystems, USA). Target lncRNAs and mRNAs were analyzed by SRAMP (http://www.cuilab.cn/sramp) to identify ACA motifs and m^6^A modification sites [47, 48]. The primers were designed using Primer 5.0 (Supplementary Table 6). Relative expression levels were calculated using the 2^-ΔΔCt^ method, and the test genes were calibrated with MazF- as follows:

%MazF-=(2^ − CtMazF+)/(2^ − CtMazF−)×100%

The experiments were carried out three times in independent determinations.

### Competing endogenous RNA network construction and functional enrichment analysis

Candidate lncRNAs verified by m^6^A single-base site qPCR were analyzed for ceRNA network construction using a previously described protocol [49]. Differentially m^6^A-methylated mRNAs as well as target mRNAs predicted by the ceRNA network were classified into GO terms based on the GO database (http://www.geneontology.org). The KEGG (http://www.genome.jp/kegg) database was also interrogated to determine the biochemical pathways enriched by these mRNAs. Hierarchical clustering was performed using R software.

### Analysis of m^6^A methylation regulators

To verify the expression of m^6^A writer, eraser, and reader proteins, qPCR experiments were performed as previously described [50]. We selected 6 representative proteins involved in m^6^A modification and binding, for which primers sequences are listed in Supplementary Table 7. The experiments were carried out three times in independent determinations.

### Statistical analysis

For qRT-PCR, microarray, and m^6^A single-base site qPCR data, differences in transcript expression and methylation levels between the LPS and control groups were evaluated using unpaired, two-sided t-test. Fisher’s exact test was applied to evaluate the significance of the GO terms and KEGG pathway identifiers for mRNAs with differential methylation levels as well as for mRNAs predicted by the ceRNA network. The analysis was performed using the limma package on R software, with the recommended cut off of *P* < 0.05.

### Data availability statement

The data that support the findings of this study are openly available in the GenBank database under accession number GSE158943 (https://www.ncbi.nlm.nih.gov/geo/query/acc.cgi?acc=GSE158943).

## Supplementary Material

Supplementary Tables 1 and 2

Supplementary Table 3

Supplementary Table 4

Supplementary Tables 5-7
