# Supplementary material for: Genome-wide identification of altered RNA m6A profiles in vascular tissue of septic rats
Source: Aging (Albany NY). 2021 Sep 10;13(17):21610–27. doi: 10.18632/aging.203506 (PMC8457599; doi:10.18632/aging.203506)
Supplement: Supplementary Tables 1 and 2 [file aging-13-203506-s001.pdf]

SUPPLEMENTARY TABLES

Supplementary Table 1. Representative lncRNAs with hyper and hypo methylated levels detected from the microarray.

Gene symbol	Type	Transcript_ID	Regulation	Fold change	P-value
Meaf6	lncRNA	XR_354272	hyper	1.966019111	0.01467847
LOC103693543	lncRNA	XR_595034	hyper	1.835972714	0.000829838
LOC102555010	lncRNA	XR_340306	hyper	1.688267079	0.01523474
AABR07039229.2	lncRNA	ENSRNOT00000074259	hyper	1.682498671	0.048551969
LOC102556135	lncRNA	XR_350748	hyper	1.658944828	0.000797307
AABR07021402.1	lncRNA	ENSRNOT00000090174	hyper	1.626112636	0.019536885
uc.330	lncRNA	uc.330+	hyper	1.616793536	0.016221101
LOC102549661	lncRNA	XR_352581	hyper	1.577837212	0.011426804
LOC103692564	lncRNA	XR_592894	hyper	1.510683227	0.000297507
LOC102556412	lncRNA	XR_348274	hypo	0.370266005	0.049209238
LOC102546594	lncRNA	XR_348260	hypo	0.432740801	0.001772264
LOC103690224	lncRNA	XR_593937	hypo	0.436711298	0.000951609
LOC102552157	lncRNA	XR_590526	hypo	0.45356657	0.002292597
AABR07019437.5	lncRNA	ENSRNOT00000087221	hypo	0.480443241	0.000178429
AC119007.3	lncRNA	ENSRNOT00000087227	hypo	0.486092398	0.008012194
AABR07064635.1	lncRNA	ENSRNOT00000089798	hypo	0.495855561	0.00151292
LOC690414	lncRNA	ENSRNOT00000080985	hypo	0.514737661	0.002069805
LOC102551164	lncRNA	XR_345350	hypo	0.526489467	0.001429385
LOC102549203	lncRNA	XR_589036	hypo	0.53133633	0.002272893

Supplementary Table 2. Top 10 of mRNAs with hyper and hypo methylated levels detected from the microarray.

Gene symbol	Type	Transcript_ID	Regulation	Fold change	P-value
Tnfrsf26	protein_coding	ENSRNOT00000066943	hyper	2.335986528	0.013354003
LOC680875	protein_coding	ENSRNOT00000014191	hyper	2.282145316	0.000014416
LOC498265	protein_coding	ENSRNOT00000087487	hyper	1.947873812	0.010762036
Camkk2	protein_coding	NM_031338	hyper	1.873342652	0.000250081
Bglap	protein_coding	ENSRNOT00000026530	hyper	1.744746614	0.029775398
Card9	protein_coding	ENSRNOT00000091484	hyper	1.742329345	0.001289323
Camkk2	protein_coding	ENSRNOT00000001774	hyper	1.732207697	0.000690433
Cnga4	protein_coding	ENSRNOT00000023751	hyper	1.728732275	0.00227076
Ptk2b	protein_coding	ENSRNOT00000030007	hyper	1.7237998	0.00437514
F12	protein_coding	ENSRNOT00000081920	hypo	1.716093098	0.005435311
RatNP-3b	protein_coding	ENSRNOT00000086035	hypo	0.220025431	0.027104865
Np4	protein_coding	ENSRNOT00000035128	hypo	0.234536087	0.011583228
Ier3	protein_coding	ENSRNOT00000080822	hypo	0.295087289	0.04831935
Slpil3	protein_coding	ENSRNOT00000076624	hypo	0.301422747	0.015878463
Slpi	protein_coding	NM_053372	hypo	0.336563105	0.017869355
Zscan25	protein_coding	ENSRNOT00000075888	hypo	0.336781044	0.032036893
Hpx	protein_coding	ENSRNOT00000024710	hypo	0.361684614	0.000014263
Reg1a	protein_coding	ENSRNOT00000057869	hypo	0.36352292	0.001119911
Nr1i2	protein_coding	ENSRNOT00000003934	hypo	0.373077429	0.000243927
Fabp1	protein_coding	ENSRNOT00000008840	hypo	0.38156521	0.035722264
