# Supplementary material for: Genome-wide identification of altered RNA m6A profiles in vascular tissue of septic rats
Source: Aging (Albany NY). 2021 Sep 10;13(17):21610–27. doi: 10.18632/aging.203506 (PMC8457599; doi:10.18632/aging.203506)
Supplement: Supplementary Table 4 [file aging-13-203506-s003.docx]

**Supplementary Table 4.** Top30 GO terms of GO analysis on hypomethylated mRNAs.

| **hypo_LPS_vs_Ctrl Methylation** | | | | | |
| --- | --- | --- | --- | --- | --- |
| **GO ID** | **Term** | **Ontology** | **P-value** | **Enrichment Score** | **Genes** |
| GO:0044419 | interspecies interaction between organisms | Biological process | 3.54323E-05 | 4.450600118 | MMP9//NP4//RATNP-3B//CAMP//SLPI//PDCD6IP//CCL3//REG1A//IFITM6//RSAD2//STAT1//SLC22A5 |
| GO:0035821 | modification of morphology or physiology of other organism | Biological process | 7.55571E-05 | 4.121724481 | MMP9//NP4//RATNP-3B//CCL3//REG1A//CAMP//SLPI |
| GO:0071674 | mononuclear cell migration | Biological process | 0.000170069 | 3.769374407 | CCL3//CCL20//CXCL17//CCL27//PDGFD |
| GO:0071222 | cellular response to lipopolysaccharide | Biological process | 0.000264222 | 3.578030696 | STAT1//CCL3//NP4//CCL20//CAMP//DEFA10//MMP9//NR1I2 |
| GO:0015711 | organic anion transport | Biological process | 0.000330144 | 3.481296903 | SLC6A18//SLC7A7//LOC292543//GRIK1//SLC26A7//SLC51B//SLC17A3//SLC27A5//FABP1//SLC22A5//SLC25A38 |
| GO:0071219 | cellular response to molecule of bacterial origin | Biological process | 0.000352968 | 3.452264428 | STAT1//CCL3//NP4//CCL20//CAMP//DEFA10//MMP9//NR1I2 |
| GO:0046942 | carboxylic acid transport | Biological process | 0.000416204 | 3.380694025 | SLC6A18//SLC7A7//LOC292543//GRIK1//SLC51B//SLC27A5//FABP1//SLC26A7//SLC25A38 |
| GO:0015849 | organic acid transport | Biological process | 0.000426167 | 3.370419687 | SLC6A18//SLC7A7//LOC292543//GRIK1//SLC51B//SLC27A5//FABP1//SLC26A7//SLC25A38 |
| GO:0002687 | positive regulation of leukocyte migration | Biological process | 0.000548646 | 3.260707733 | CXCL17//CCL27//CCL20//PDGFD//CCL3//MMP9 |
| GO:0002376 | immune system process | Biological process | 0.000562148 | 3.250149058 | CTSH//NP4//CAMP//BPIFB1//STAT1//IL22//CCL3//CCL20//TAPBPL//HPX//MMP9//CLEC4G//IL1F10//CCL27//LST1//SLPI//CXCL17//AHSP//SLC25A38//DYRK3//VAV3//RSAD2//IFITM6//IL18RAP//IL18BP//FANCD2//BPGM//OLFM4//REG1A//PDGFD |
| GO:0005615 | extracellular space | Cellular component | 5.76158E-05 | 4.239458095 | PDCD6IP//LOC100909605//GC//REG1A//CTSH//CCL3//NP4//OLFM4//LTBP4//CCL20//SLURP1//CXCL17//RGD1308195//CAMP//IL1F10//LEFTY1//RATNP-3B//IL22//HPX//DEFA10//PDGFD//MMP9//SLPI//IL18BP |
| GO:0005576 | extracellular region | Cellular component | 0.000400471 | 3.397429083 | LTBP4//MMP9//LOC100909605//GC//REG1A//CTSH//CCL3//NP4//OLFM4//CCL20//SLURP1//CXCL17//RGD1308195//CAMP//IL1F10//LEFTY1//RATNP-3B//IL22//HPX//DEFA10//PDGFD//SLPI//IL18BP//PDCD6IP//WFDC18//CCL27//SLPIL3//BPIFB1 |
| GO:0044421 | extracellular region part | Cellular component | 0.000580644 | 3.236090205 | LOC100909605//GC//REG1A//CTSH//CCL3//NP4//OLFM4//LTBP4//CCL20//SLURP1//CXCL17//RGD1308195//CAMP//IL1F10//LEFTY1//RATNP-3B//IL22//HPX//DEFA10//PDGFD//MMP9//SLPI//IL18BP//PDCD6IP |
| GO:0005811 | lipid droplet | Cellular component | 0.002293485 | 2.639504035 | PNPLA5//METTL7B//PLIN5//RSAD2 |
| GO:0042581 | specific granule | Cellular component | 0.004401048 | 2.356443857 | OLFM4//CAMP |
| GO:0032809 | neuronal cell body membrane | Cellular component | 0.022053119 | 1.656529971 | ATP2B2//REG1A |
| GO:0044298 | cell body membrane | Cellular component | 0.023367062 | 1.631395882 | ATP2B2//REG1A |
| GO:0098839 | postsynaptic density membrane | Cellular component | 0.023367062 | 1.631395882 | ATP2B2//GRIK1 |
| GO:0099634 | postsynaptic specialization membrane | Cellular component | 0.028932341 | 1.538616431 | ATP2B2//GRIK1 |
| GO:0016323 | basolateral plasma membrane | Cellular component | 0.033425573 | 1.475921142 | SLC27A5//SLC22A5//SLC26A7//SLC51B//SLC7A7 |
| GO:0008514 | organic anion transmembrane transporter activity | Molecular function | 2.14E-05 | 4.669771869 | LOC292543//SLC26A7//SLC51B//SLC17A3//SLC7A7//SLC6A18//SLC25A38//SLC27A5//SLC22A5 |
| GO:0008509 | anion transmembrane transporter activity | Molecular function | 7.07E-05 | 4.150823016 | LOC292543//SLC26A7//GLRA3//SLC22A5//SLC17A3//SLC51B//SLC7A7//SLC6A18//SLC25A38//SLC27A5 |
| GO:0005342 | organic acid transmembrane transporter activity | Molecular function | 8.46E-05 | 4.072447892 | LOC292543//SLC51B//SLC7A7//SLC6A18//SLC25A38//SLC27A5//SLC26A7 |
| GO:0046943 | carboxylic acid transmembrane transporter activity | Molecular function | 8.46E-05 | 4.072447892 | LOC292543//SLC51B//SLC7A7//SLC6A18//SLC25A38//SLC27A5//SLC26A7 |
| GO:0048020 | CCR chemokine receptor binding | Molecular function | 0.000143366 | 3.843553316 | CCL3//CCL27//STAT1//CCL20 |
| GO:0015318 | inorganic molecular entity transmembrane transporter activity | Molecular function | 0.000156705 | 3.804915944 | LOC292543//GRIK1//GLRA3//KCNG3//SLC26A7//SLC24A2//SLC6A18//ATP2B2//SLC17A3//SLC22A5//ATP6V1G2//SLC51B//SLC7A7//SLC25A38//SLC27A5//SLC9A8 |
| GO:0015297 | antiporter activity | Molecular function | 0.000196831 | 3.705906153 | SLC24A2//SLC26A7//SLC9A8//SLC22A5//SLC7A7 |
| GO:0015075 | ion transmembrane transporter activity | Molecular function | 0.000274963 | 3.560725338 | LOC292543//GRIK1//GLRA3//KCNG3//SLC26A7//SLC24A2//SLC6A18//ATP2B2//SLC17A3//SLC22A5//ATP6V1G2//SLC51B//SLC7A7//SLC25A38//SLC27A5//SLC9A8 |
| GO:0022857 | transmembrane transporter activity | Molecular function | 0.000281263 | 3.550887697 | LOC292543//GRIK1//GLRA3//KCNG3//SLC26A7//SLC24A2//SLC6A18//ATP2B2//SLC17A3//SLC22A5//ATP6V1G2//SLC51B//SLC7A7//SLC25A38//HPX//SLC27A5//SLC9A8//GC |
| GO:0099516 | ion antiporter activity | Molecular function | 0.00050933 | 3.293000759 | SLC24A2//SLC26A7//SLC9A8//SLC22A5 |
