# Supplementary material for: Genome-wide identification of altered RNA m6A profiles in vascular tissue of septic rats
Source: Aging (Albany NY). 2021 Sep 10;13(17):21610–27. doi: 10.18632/aging.203506 (PMC8457599; doi:10.18632/aging.203506)
Supplement: Supplementary Tables 5-7 [file aging-13-203506-s004.pdf]

Supplementary Table 5. The detailed information of enriched pathway on hyper and hypomethylated mRNAs.

Pathway ID	Definition	Regulation	Fisher-P value	Enrichment score	Genes
rno04610	Complement and coagulation cascades	hyper	1.85625E-05	4.731363	BDKRB1//F12//KNG1//SERPING1
rno04750	Inflammatory mediator regulation of TRP channels	hyper	0.0230158	1.637974	BDKRB1//KNG1
rno04080	Neuroactive ligand-receptor interaction	hyper	0.03544862	1.450401	BDKRB1//KNG1//LTB4R
rno04062	Chemokine signaling pathway	hypo	0.001848041	2.733288	CCL20//CCL27//CCL3//ELMO1//STAT1//VAV3
rno04060	Cytokine-cytokine receptor interaction		0.003188302	2.496441	CCL20//CCL27//CCL3//CXCL17//IL18RAP//IL1F10//IL22
rno04061	Viral protein interaction with cytokine and cytokine receptor		0.003329375	2.477637	CCL20//CCL27//CCL3//IL18RAP
rno05150	Staphylococcus aureus infection		0.005536582	2.256758	CAMP//DEFA10//NP4//RATNP-3B
rno04621	NOD-like receptor signaling pathway		0.0102476	1.989378	CAMP//DEFA10//NP4//RATNP-3B//STAT1
rno05321	Inflammatory bowel disease (IBD)		0.01216042	1.915051	IL18RAP//IL22//STAT1
rno00062	Fatty acid elongation		0.02118827	1.673904	ELOVL1//PPT2
rno03320	PPAR signaling pathway		0.02309338	1.636513	FABP1//PLIN5//SLC27A5
rno05323	Rheumatoid arthritis		0.03087388	1.510409	ATP6V1G2//CCL20//CCL3

Supplementary Table 6. Sequence of primers used for m^6A single-base site qPCR analysis of lncRNAs and mRNAs methylation levels.

Gene names	Type	Sequence	Product size (bp)
XR_595701	lncRNA	F:5’ GCTGAGCAAAGGTGCCACT 3’<br>R:5’ GAAGCCGCCATCTTTCATCT3’	99
XR_343955	lncRNA	F:5’ TTTCCTAACGAGGCTCACAG3’<br>R:5’ ATTGGAATTGGTAGGGTATCG3’	153
XR_593937	lncRNA	F:5’ CCAGCAGATGGGATGATTT3’<br>R:5’ AGAAGTCCAAGGATCAGGGT3’	193
XR_595034	lncRNA	F:5’ ATCTCATCCTGCCGCTCCTT3’<br>R:5’ TCTTCCGCCTCCAGCACTTA 3’	276
XR_353597	lncRNA	F:5’ GGGCTCTGAACCAGTACCAAA3’<br>R:5’ TCCAGGAGAAGGGCATCCTT3’	289
ENSRNOT00000012927	mRNA	F:5’ TTCTCAGAAGCCAGAGTTAGAGTC3’<br>R:5’ GGATAACCTGTGCAGGTGTTG3’	192
ENSRNOT00000078131	mRNA	F:5’ CTTAGCAATGGACACCAGAAA3’<br>R:5’ ACCATGACCAAGACCATAACC3’	141
ENSRNOT00000066943	mRNA	F:5’ GCTGTCCTGAGGGCAGAGTC3’<br>R:5’ TGACGGGAGTAGCGAATGAA3’	184
ENSRNOT00000010760	mRNA	F:5’ CCTTTCCTCCGTGAAGACTGT3’<br>R:5’ CAATTGTGTTATGAATATCCACGTA3’	91
ENSRNOT00000030109	mRNA	F:5’ GCCAGGCTATCTGAGCGACA3’<br>R:5’ GAGAGGTCGGCGGTGGTAGT3’	155

Supplementary Table 7. Sequence of primers used for qRT-PCR analysis of mRNA levels.

Gene names	Sequence	Product Size (bp)
GAPDH(RAT)	F:5’ GCTCTCTGCTCCTCCCTGTTCTA3'<br>R:5’ TGGTAACCAGGCGTCCGATA3’	124
METTL3	F:5’ TTGACTACAGTGGCTACCTTT3’<br>R:5’ CCTTGGCTGTTGTGGTATT3’	220
METTL14	F:5’ GAGTATGTTTGCGAAAGTGGG3’<br>R:5’ TTGTCTTTCCAGGATTGTTCTT 3’	84
WTAP	F:5’ GAAAAACTAAAGCAGCAACAG3’<br>R:5’ CGTAAACTTCCAGGCACTC3’	267
YTHDF1	F:5’ GCCAGGAGGAAGAGGAGGTA 3’<br>R:5’ AGACAGCACCAAGCATACAGC 3’	131
YTHDF3	F:5’ GCCATGCGAAGGGAGAGAA3’<br>R:5’ AGCTTCAGGACACAAAGTGCT3’	278
FTO	F:5’ GAGCGGGAAGCTAAGAAA 3’<br>R:5’ GCTGCCACTGCTGATAGAA 3’	100

METTL3, methyltransferase like 3; METTL14, methyltransferase like 14; WTAP, Wilms-tumour-1 associated protein; YTHDF1, YT521-B homology domain family 1; YTHDF3, YT521-B homology domain family 3; FTO, fat mass and obesity-associated protein.
